# Physiotherapy Compared With Shockwave Therapy for the Treatment of Proximal Hamstring Tendinopathy: A Randomized Controlled Trial

**DOI:** 10.1177/03635465251391134

**Published:** 2025-11-16

**Authors:** Aidan Rich, Jon Ford, Jilliane Cook, Andrew Hahne

**Affiliations:** † School of Allied Health, Human Services and Sport, La Trobe University, Bundoora, Victoria, Australia; ‡ Lifecare Malvern Sports Medicine, Malvern, Victoria, Australia; § Advance Healthcare, Boronia, Victoria, Australia; Investigation performed at La Trobe University, Bundoora, Victoria, Australia

**Keywords:** tendinosis, hip/pelvis/thigh, running, physical therapy/rehabilitation, shockwave therapy

## Abstract

**Background:**

Proximal hamstring tendinopathy (PHT) presents as localized lower buttock pain with tasks such as running and sitting.

**Purpose:**

To investigate the effectiveness of individualized physiotherapy compared with shockwave therapy on pain and function in PHT.

**Methods:**

This prospective parallel-group, assessor-blinded randomized controlled trial (RCT) was set in 10 primary care physiotherapy practices in Victoria, Australia. A total of 100 participants with PHT were randomly assigned to receive 6 sessions of either individualized physiotherapy or shockwave therapy, with both groups receiving standardized advice and education. Primary outcomes were global rating of change on a 7-point Likert scale and the Victorian Institute of Sport Assessment Scale for Proximal Hamstring Tendinopathy at 4, 12, 26, and 52 weeks postrandomization. Analyses were by intention-to-treat using linear mixed models.

**Results:**

All except 1 patient received the allocated intervention, and the participant follow-up rate was 88% at 12 months. There were no significant differences between groups in primary outcomes at any time point. For secondary outcome measures, participants in the shockwave group had statistically significantly greater satisfaction with treatment at 26 weeks, satisfaction with results of treatment at 4 and 26 weeks, and general health at 52 weeks. Responder analyses of participants achieving clinically significant improvements showed no significant differences between groups at any time point.

**Conclusion:**

This RCT found no difference in effectiveness of individualized physiotherapy compared with shockwave therapy on global effect or function in PHT. Future trials on PHT appear feasible but could explore a different sample population or other intervention and/or comparison groups.

**Registration:**

ACTRN12621000846820 (Australian New Zealand Clinical Trials Registry).

Proximal hamstring tendinopathy (PHT) primarily affects active individuals, particularly those engaging in running, lunging, or kicking sports. Initially described in 1988 as “hamstring syndrome,”^77^ PHT is characterized by focal pain in the lower buttock region, exacerbated by activities such as prolonged sitting, running, lunging, squatting, and walking.^10,34,50^

Tendinopathy is attributed to periods of acute and/or chronic overloading or unloading of the tendon, encompassing both tensile and compressive loads. Acute overloading can result in reactive tendinopathy,^15^ manifesting as tendon swelling caused by increased large proteoglycan content without disruption of collagen structure. Chronic overloading, however, can lead to disrepair or degenerative^15^ tendinopathy characterized by collagen and tendon matrix disorganization.

Although tendons primarily transmit tensile forces, compressive forces are recognized as contributors to adaptive changes in the tendon matrix and the development of pathology.^87^ In PHT, compressive loading typically occurs at the ischial tuberosity, particularly during activities involving deep hip flexion, such as squatting, lunging, and kicking, as well as when sitting, or when stretching the hamstrings. Such compressive loads are commonly provocative in individuals with PHT.^34,50^ Surgical^51^ and imaging^100^ studies demonstrate tendinopathic changes near the ischial tuberosity, supporting the role of compression as a significant factor in the development of PHT.

Research on the diagnostic criteria for PHT remains sparse, with no universally accepted diagnostic gold standard. While 1 study investigated the diagnostic accuracy of 3 stretching tests using magnetic resonance imaging (MRI) as the reference standard, its conclusions were limited by the prevalence of abnormal proximal hamstring findings in asymptomatic individuals.^18^ Despite these limitations, there is consensus on key clinical indicators of PHT, including pain provocation during tensile and/or compressive loading of the tendon, such as during muscle contraction, functional tasks, stretching, or sitting,^34,50,59,77^ and focal pain localized over the proximal tendon.^29,37,58,64,78^ An additional characteristic often is a history of increased tendon load before symptom onset.^34,59^

A range of treatment options have been proposed for PHT. Nonoperative approaches traditionally include load management, graded exercise rehabilitation, the selective use of oral nonsteroidal anti-inflammatory drugs, and manual therapy techniques.^34,50^ Injection therapies, such as platelet-rich plasma, autologous blood, and corticosteroids, have also been proposed.^16,100^ However, there is insufficient high-quality evidence supporting the effectiveness of these treatments specifically for PHT.^16,22,53,70,98,100^

Extracorporeal shockwave therapy (ESWT), a noninvasive treatment, has shown promise in other forms of tendinopathy.^31,49,54,81,88^ Some mechanisms proposed to explain its treatment effect include reduced pain pressure thresholds^52,93^ and alterations in tendon collagen structure,^7,52^ although these findings are largely limited to studies of other tendons or animal models. A previous randomized controlled trial (RCT) compared ESWT with nonoperative care for PHT,^10^ reporting superior results for pain and function at the 3-month follow-up. However, the trial had notable limitations, including a nonindividualized nonoperative care program and a lack of alignment with established protocols for successfully treating other tendinopathies.^68^

Emerging recommendations have highlighted the importance of individualized rehabilitation for PHT, emphasizing progressive strengthening exercises, gradual reintroduction of compressive loads, restoration of tendon energy storage and release capacity, and graded return to normal activities.^34,59^ Progressive strengthening programs have demonstrated efficacy in improving pain and function for other lower limb tendinopathies,^∥^ but their application to PHT has not been thoroughly evaluated, particularly in protocols tailored to individual presentations.^39,42,72^

Given these gaps in the literature, further research is warranted to evaluate the effectiveness of individualized physiotherapy for individuals with clinical features indicative of PHT. This RCT aimed to address this need by comparing individualized physiotherapy (PHYSIOTHERAPY) with ESWT (SHOCKWAVE) in terms of pain, strength, and function outcomes. Additionally, this trial aimed to evaluate the feasibility of recruiting and following up individuals with PHT.

## Methods

### Trial Design

This multicenter RCT was registered prospectively in the Australian New Zealand Clinical Trials Registry (ACTRN12621000846820), and the protocol was published.^79^ Ethical approval was obtained (La Trobe University Human Ethics Committee [HEC21049]). Participants were given a written information and consent form, which was signed before enrollment in the trial. Patients and/or the public were not involved in the design, conduct, reporting, or dissemination plans of this research.

### Participants and Recruitment

Participants were recruited from public and social media advertising and referrals from physiotherapists, sport and exercise medicine physicians, and orthopaedic surgeons. Eligibility criteria included age of 18 to 65 years and a ≥3-month history of gradual onset of localized lower buttock pain, with clinical features of PHT including a history of increased tendon load precipitating the onset of symptoms, and pain on ≥3 of 4 loading/compressive tests.^79^

Eligible participants were required to have no history of previous hamstring surgery, no injections to the hamstring tendon in the previous 6 weeks, and no shockwave to the hamstring tendon in the previous 3 months, as we wished to study the effects of intervention independent of the effects of these treatments. Full eligibility criteria are listed in Appendix Table A1 (available in the online version of this article).

### Randomization and Allocation Concealment

Participants were randomly allocated into 1 of 2 intervention groups: PHYSIOTHERAPY or SHOCKWAVE. A randomization schedule was prepared ahead of time by a researcher (A.H.) at La Trobe University, located remotely from the treatment sites. The randomization sequence was generated electronically using an online randomization program and incorporated random block sizes. Stratification for age (<50 years vs ≥50 years) was undertaken due to the hypothesis that systemic factors associated with menopause may affect response to some aspects of intervention.^5,11,20,91^

To ensure concealed allocation of participants to groups in accordance with the off-site randomization schedule, the primary researcher (A.R.) emailed each consenting participant’s name and date of birth to the La Trobe University researcher; the participant was entered into the trial and their treatment allocation and randomization date returned to the primary researcher via reply email. The primary researcher then provided the treating physiotherapist with the intervention allocation and contact details to arrange an initial appointment.

### Interventions

Intervention protocols for both groups were outlined in a comprehensive treatment manual, complemented by a digital clinical notes template. The template provided guidance for intervention consistent with the trial protocol for all treatment sessions. Intervention was matched for time and session exposure to the physiotherapist. Both groups received 6 sessions of treatment (the first session, 1 hour; the remainder, 30 minutes) over a 12-week period, at 0, 1, 2, 3, 6, and 12 weeks after randomization.

#### SHOCKWAVE Intervention

Intervention for SHOCKWAVE participants followed the trial approach in Cacchio et al,^10^ with 4 sessions of ESWT provided at weekly intervals. The protocol was standardized with participants receiving 2000 shocks at the maximum tolerable intensity, consistent with safe and effective dosages in previous trials.^84^ No ESWT was provided in the final 2 sessions; these were used to review relevant information sheets and plan return to normal activity. Participants allocated to the SHOCKWAVE intervention were not prescribed any exercise by their physiotherapist and were asked to refrain from seeking other treatments during the trial.

Both radial and semifocused shockwave devices were used in this trial, as previous research has found no difference in outcomes between the 2 in the treatment of tendinopathy.^83,84^

#### PHYSIOTHERAPY Intervention

Intervention for PHYSIOTHERAPY participants was based on treatment with demonstrated effectiveness in other lower limb tendinopathies,^48,86^ and pertaining to known or hypothesized mechanisms underpinning PHT. The full intervention protocol has been submitted for publication. A fundamental aspect of the program was an individualized, multistage, progressive rehabilitation/strengthening program. A graded reintroduction of compressive forces to the proximal hamstring was incorporated into the intervention algorithms, consistent with expert recommendations for PHT^69^ and other tendinopathies.^13,14,36,65^

#### Participant Education and Other Co-interventions

Participants in both groups received standardized information sheets prepared specifically for the trial, covering topics such as diagnosis, treatment options, expected recovery time frames, monitoring of pain, the role of compression (including sitting) in tendinopathy, and high and low tendon loading activities. Known or hypothesized mechanisms underlying treatment of this condition informed the content on the information sheets. Participants were able to continue their normal activities (including running) on a pain-contingent basis, with up to 4 of 10 pain or “moderate discomfort” allowed during activity, and no significant (>2/10) increase in pain in the 12 to 24 hours after activity. Participants could continue with any medication for their condition. In addition, treating physiotherapists were able to refer participants to a pharmacist or their general practitioner during the treatment program if they determined that medication review was warranted, although medication was not part of the standard treatment in either intervention.

### Blinding

Blinding of participants and treating physiotherapists was not possible due to the nature of the interventions, although treating practitioners and the primary researcher informed participants that both treatment approaches had a realistic chance of improving function and symptoms and that neither had previously been shown to be superior. Training of physiotherapists emphasized the treatment of participants receiving either intervention with identical expectation and enthusiasm. Strength outcome measures were undertaken by an assessor blinded to treatment allocation.

### Treating Physiotherapists and Treatment Fidelity

Treatment for both groups was provided by physiotherapists from private practices in Melbourne and Ballarat, Australia. At least 2 years of clinical experience was required, and physiotherapists participated in an initial small-group, half-day training session facilitated by the primary researcher (A.R.). The training session covered previously provided material and practical simulation of treatments and explanations utilized in the trial.

Treating physiotherapists were provided with a treatment manual detailing treatment algorithms, protocols, and participant information sheets. The treatments were standardized and specified utilizing detailed electronic clinical note templates, which included decision-making algorithms. The combination of clinical notes and algorithms was designed to ensure that vital components of the treatment program were consistently applied for all treatment sessions, but still allowing some individualization of treatment around the presentation of individual participants. On the templates, the treating physiotherapists were required to input physical assessment findings, justification and rationale for clinical decision-making, details on the treatment provision/prescription, and response to treatment.

Evaluation of treatment fidelity was undertaken by the primary researcher by review of the physiotherapist’s clinical notes after sessions 2, 4, and 6 of treatment for each participant.

### Outcome Measures

Outcome measures are shown in Table 1. Primary outcomes were global rating of change (GROC), measured using a 7-point Likert scale,^6,46^ and the Victorian Institute of Sport Assessment Scale for Proximal Hamstring Tendinopathy (VISA-H) questionnaire.^9^ Both primary outcome measures have been shown to be valid, reliable, and responsive.^9,46,47^ All secondary outcomes have good reliability and validity.^¶^

**Table 1:** Outcome Measures

Outcome Measure	Measurement Point, wk
Primary outcome measures	
1. Global rating of change scale (7-point Likert scale)	4, 12, 26, 52
2. VISA-H (8-item questionnaire)	0, 4, 12, 26, 52
Secondary outcome measures	
1. Sitting tolerance (5-point scale)	0, 4, 12, 26, 52
2. Modified Physical Activity Level Scale (6-point scale)	0, 4, 12, 26, 52
3. Eccentric hamstring strength, N	0, 12
4. Modified Tampa Scale for Kinesiophobia (TSK-11) (11-item questionnaire)	0, 4, 12, 26, 52
5. Örebro Musculoskeletal Pain Screening Questionnaire–Short Form (ÖMPSQ-SF)	0, 4, 12, 26, 52
6. Pain Catastrophizing Scale (PCS)	0, 4, 12, 26, 52
7. Numeric pain rating scale (average and most severe pain over previous week)	0, 4, 12, 26, 52
8. Participant rating of adherence (11-point Likert scale)	4, 12, 26, 52
9. Satisfaction with treatment (5-point Likert scale)	4, 12, 26, 52
10. Satisfaction with the results of treatment (5-point Likert scale)	4, 12, 26, 52
11. EQ-5D	0, 4, 12, 26, 52

All outcomes were assessed through self-administered electronic questionnaires on QuestionPro, with the exception of strength, which was measured by a blinded assessor. Links to QuestionPro were emailed to participants at the relevant time points (Table 1). Outcome measures were consistent with consensus guidelines for tendinopathy health domains^94^ and participant reporting characteristics^80^ (for baseline data). Feasibility was measured with recruitment rate, participant retention, and outcome measure completion rates.

### Sample Size

Given the absence of robust data for the VISA-H or any other outcome measures for PHT on which to calculate sample size, 100 was pragmatically chosen to determine the feasibility of any future trials on this population and assist with an accurate sample size calculation. The chosen sample size of 100 provided 80% power to detect a between-group standardized mean difference of at least 0.6 on continuous outcome measures (eg, the VISA-H), allowing for 10% loss to follow-up.^55^ Smaller effect sizes may still be considered clinically significant, so the trial was not fully powered to detect all potentially important effects.

### Feasibility

We aimed for a recruitment rate of 12 participants per month, and participant retention and outcome measure completion >85%, consistent with PEDro guidelines.^57^

### Statistical Analysis

Data from all follow-up points (Table 2) were analyzed after the conclusion of the trial, with emphasis on estimating between-group treatment effects (with 95% confidence intervals). Alpha was set at .05 using a 2-tailed hypothesis and SPSS (Version 30) (IBM) was used for conducting analyses.

**Table 2:** Baseline Participant and Clinical Characteristics*^a^*

Characteristic	SHOCKWAVE (n = 50)	PHYSIOTHERAPY (n = 50)
Demographics		
Age, y	45.4 (11.6)	44.4 (9.4)
Female sex	37 (74)	37 (74)
Duration of symptoms, wk	92.3 (110.4)	125.5 (207.3)
Left side affected	23 (46)	26 (52)
Anthropometrics		
Weight, kg*^b^*	69.5 (11.6)	62.5 (8.1)
Height, cm*^b^*	170.8 (8.0)	169.4 (8.1)
Body mass index, kg/m^2^	23.4 (2.8)	22.4 (2.5)
Nicotine use	00 (0)	00 (0)
Primary outcome measures		
VISA-H	49.8 (15.4)	50.7 (11.8)
Secondary outcome measures		
Kinesiophobia (TSK-11)	22.6 (5.8)	22.8 (4.8)
Catastrophization (PCS)	11.7 (10.8)	11.9 (9.5)
ÖMPSQ-SF	35.9 (10.8)	37.9 (10.4)
Average pain over last week on NRS	4 (2.0)	4.18 (1.5)
Maximum pain over last week on NRS	5.5 (2.2)	5.9 (1.8)
Quality of life (EQ-5D-5L utility score)	0.76 (0.08)	0.75 (0.10)
General health (EQ-5D-5L thermometer)	81.5 (9.5)	82.1 (9.8)
Strength, N	207.8 (71.9)	214.9 (60)
Physical Activity Level Scale	5.5 (5.0-6.0)	5.0 (4.0-6.0)
Sitting symptoms	2.0 (1.0-3.0)	2.0 (1.0-3.0)

a Data are presented as n (%), mean (SD), or median (25th-75th percentile). NRS, numeric rating scale (scored from 0 to 10); ÖMPSQ-SF, Örebro Musculoskeletal Pain Screening Questionnaire–Short Form; PCS, Pain Catastrophizing Scale; TSK-11, Tampa Scale for Kinesiophobia; VISA-H, Victorian Institute of Sport Assessment Scale for Proximal Hamstring Tendinopathy.

b Self-reported.

Intention-to-treat principles were used for all analyses, with participants analyzed on the basis of their original allocation regardless of the number of sessions attended or treatment adherence.^43^ Maximum likelihood estimation within linear mixed models was used to manage missing data.^27^

Linear mixed models were used for analyzing continuous data with adjustment for baseline scores of the outcome being analyzed as well as the stratification variable of age (the group × time interaction estimating the between-group treatment effect). The Mann-Whitney *U* test was used at each time point for ordinal data, acknowledging that multiple tests increase the risk of type 1 errors but would be partly offset by the lower power associated with nonparametric tests.

A responder analysis was also undertaken to assess the proportion of participants achieving clinically significant changes in outcomes. We defined the minimal clinically important difference (MCID) as 12 points on the VISA-H questionnaire^9^ and at least “much improved” on the GROC.^26,75^ The VISA-H MCID value was chosen as it is similar to values on other VISA scales.^25,41,66,76^ Risk ratio, risk difference, and number needed to treat were calculated for all responder analyses along with 95% confidence intervals,^38^ while statistical significance was determined using chi-square analysis.

### Equity, Diversity, and Inclusion Statement

Our clinical trial includes both men and women with PHT from Victoria, Australia. Our research and author team included 1 woman and 3 men, all from the same country, and 1 is a junior researcher. An additional effect modifier analysis (manuscript in preparation) explores the effect of sex; however, we did not examine the effects of race/ethnicity or socioeconomic status.

## Results

**Figure 1.**
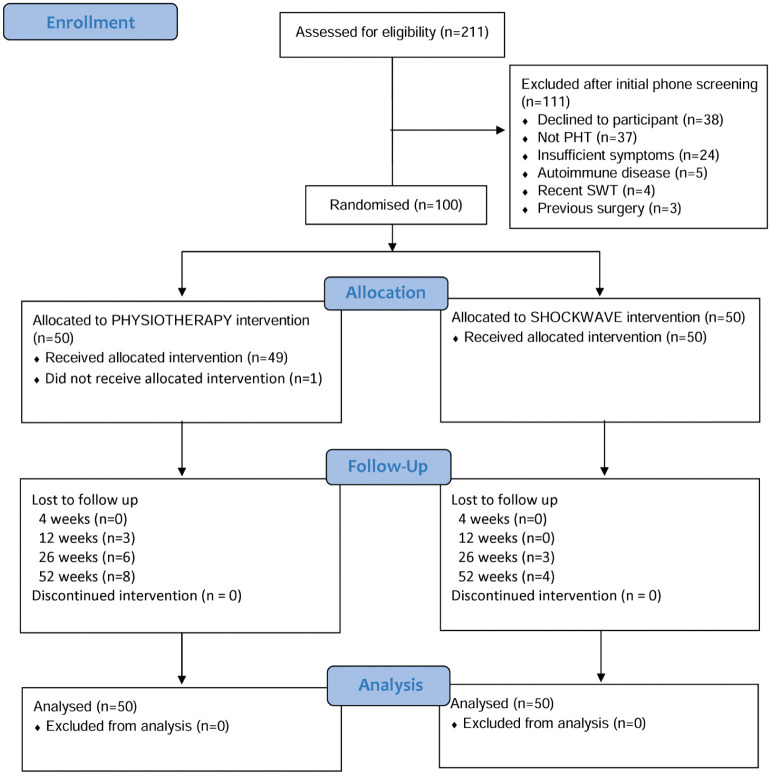
Flowchart of participant inclusion and exclusion in trial. PHT, proximal hamstring tendinopathy; SWT, shock wave therapy.

This trial recruited 100 participants between November 2021 and June 2023. Restrictions related to the COVID-19 pandemic resulted in delays of approximately 2 months. A flowchart showing participant inclusion and exclusion is presented in Figure 1. Recruitment rate was approximately 6 participants per month, and follow-up rates were 85% to 100% at 4 to 52 weeks. Groups were well matched at baseline (Table 2) with the exception of symptom duration, which was higher in the SHOCKWAVE group, but further analysis revealed that this was driven by some outliers, with a median symptom duration of 53.6 weeks in the PHYSIOTHERAPY group and 58.6 weeks in the SHOCKWAVE group. Baseline VISA-H scores were similar to those seen in other nonsurgical and surgical populations,^9^ suggesting moderate functional and pain impairments. The median Physical Activity Level Scale (PALS) scores at baseline were 5.5 for the PHYSIOTHERAPY group and 5.0 for the SHOCKWAVE group, where a score of 5 is described as “moderate exercise at least 3h/wk, e.g. tennis, swimming, jogging” and a score of 6 (the highest possible score) is described as “hard or very hard exercise regularly (several times a week in which the physical exertion is great, e.g. jogging, skiing.” Both groups reached a median score of 6 of 6 by 26 weeks. The mean (SD) number of treatment sessions attended over the 12-week intervention was 5.9 (0.2) for the SHOCKWAVE intervention and 5.6 (1.1) for the PHYSIOTHERAPY intervention. One participant randomized to receive PHYSIOTHERAPY failed to commence the intervention. There were no adverse events reported in either group.

There were no statistically significant between-group differences in the 2 primary outcome measures (VISA-H and GROC) at any time point (Tables 3 and 4).

**Table 3:** Effects of Physiotherapy Versus Shockwave on Continuous Primary and Secondary Outcomes*^a^*

Outcome	No. Included, PHYSIOTHERAPY/ SHOCKWAVE	Unadjusted Mean Score (SD)				
		PHYSIOTHERAPY	SHOCKWAVE	Adjusted Between- Group Difference (95% CI)	Adjusted SMD (95% CI)	*P* Value
Pain, function, and sporting activity (VISA-H: 8-item questionnaire scored out of 100): higher scores indicate lower pain, higher function, and sporting activity						
Baseline	50/50	49.8 (15.4)	50.7 (11.8)	—	—	—
4 wk	50/50	57.6 (16.1)	56.5 (15.5)	2.0 (–3.6 to 7.7)	0.1 (–0.3 to 0.5)	.480
12 wk	47/50	65.2 (16.9)	65.5 (16.4)	1.1 (–5.5 to 7.6)	0.1 (–0.3 to 0.5)	.746
26 wk	43/47	68.3 (17.9)	72.8 (15.1)	–3.8 (–10.4 to 2.8)	–0.2 (–0.6 to 0.2)	.252
52 wk	41/46	73.9 (18.9)	74.7 (17.9)	–0.6 (–8.1 to 6.9)	0.0 (–0.5 to 0.4)	.873
Eccentric hamstring strength, N						
Baseline	49/49	207.8 (71.9)	214.9 (60.1)	—	—	—
12 wk	44/48	225.9 (72.2)	228.4 (66.5)	8.8 (–5.4 to 23.1)	0.1 (–0.3 to 0.5)	.221
Kinesiophobia (modified Tampa Scale for Kinesiophobia [TSK-11]: 11-item questionnaire scored out of 44): higher scores indicate higher kinesiophobia						
Baseline	50/50	22.6 (5.8)	22.8 (4.8)	—	—	—
4 wk	50/50	20.7 (5.2)	20.7 (5.5)	0.2 (–1.3 to 1.7)	00 (–0.4 to 0.4)	.770
12 wk	47/50	19.0 (5.0)	19.0 (5.4)	0.1 (–1.9 to 2.0)	00 (–0.4 to 0.4)	.956
26 wk	42/47	18.9 (5.1)	18.1 (5.4)	0.9 (–1.2 to 3.0)	0.2 (–0.3 to 0.6)	.398
52 wk	41/46	19.4 (7.1)	18.3 (5.3)	1.0 (–1.9 to 3.9)	0.2 (–0.3 to (0.6)	.504
Örebro Musculoskeletal Pain Screening Questionnaire–Short Form (10 items scored out of 100): higher scores indicate greater psychosocial risk						
Baseline	50/50	35.9 (10.8)	37.9 (10.4)	—	—	—
4 wk	50/50	39.7 (16.4)	36.9 (19.5)	4.2 (–3.8 to 12.1)	0.2 (–0.2 to 0.6)	.302
12 wk	47/50	23.5 (10.1)	25.8 (10.5)	–0.3 (–4.6 to 3.9)	00 (–0.4 to 0.4)	.873
26 wk	42/47	28.1 (11.2)	25.6 (11.9)	4.7 (0.2 to 9.1)	0.4 (0.0 to 0.8)	.039*^b^*
52 wk	41/46	24.5 (11.9)	23.9 (12.2)	3.2 (–2.0 to 8.3)	0.1 (–0.4 to 0.5)	.227
Catastrophizing (Pain Catastrophizing Scale: 13-item scale scored out of 52): higher scores indicate higher catastrophizing						
Baseline	50/50	11.7 (10.8)	11.9 (9.5)	—	—	—
4 wk	50/50	7.9 (8.4)	7.6 (8.4)	0.4 (–2.4 to 3.3)	0.1 (–0.3 to 0.4)	.762
12 wk	47/50	4.9 (7.4)	5.2 (6.5)	–0.2 (–3.1 to 2.6)	00 (–0.4 to 0.4)	.870
26 wk	42/47	4.6 (5.9)	3.6 (5.7)	1.1 (–1.7 to 4.0)	0.2 (–0.2 to 0.6)	.431
52 wk	41/46	5.0 (8.7)	3.5 (5.7)	1.4 (–1.9 to 4.6)	0.2 (–0.2 to 0.6)	.411
Most severe pain over the last week (numeric pain rating scale: scored from 0 to 10)						
Baseline	50/50	5.5 (2.2)	6.0 (1.8)	—	—	—
4 wk	50/50	4.3 (2.1)	4.2 (2.3)	0.6 (–0.3 to 1.5)	0.1 (–0.3 to 0.5)	.198
12 wk	47/50	3.0 (2.3)	2.9 (1.9)	0.6 (–0.4 to 1.6)	0.1 (–0.3 to 0.5)	.229
26 wk	42/47	3.1 (2.1)	2.6 (2.2)	1.0 (–0.1 to 2.0)	0.2 (–0.3 to 0.6)	.064
52 wk	41/46	2.8 (2.5)	2.6 (2.4)	0.6 (–0.5 to 1.6)	00 (–0.4 to 0.5)	.283
Average pain over the last week (numeric pain rating scale: scored from 0 to 10)						
Baseline	50/50	4.0 (2.0)	4.2 (1.5)	—	—	—
4 wk	50/50	2.8 (1.6)	2.7 (1.8)	0.2 (–0.5 to 0.9)	0.1 (–0.3 to 0.5)	.551
12 wk	47/50	1.7 (1.4)	1.7 (1.5)	0.2 (–0.5 to 0.9)	0.1 (0.3 to 0.5)	.597
26 wk	42/47	1.8 (1.5)	1.6 (1.6)	0.5 (–0.3 to 1.2)	0.3 (–0.1 to 0.7)	.204
52 wk	41/46	1.7 (1.9)	1.8 (2.0)	0.2 (–0.7 to 1.0)	–0.1 (–0.5 to 0.4)	.700
Participant rating of adherence (11-point Likert scale): higher scores indicate greater adherence						
4 wk	49/50	7.6 (2.3)	8.5 (2.0)	–0.9 (–1.8 to –0.0)	–0.4 (–0.8 to 0.0)	.039*^b^*
12 wk	47/50	7.4 (2.3)	9.0 (1.4)	–1.6 (–2.4 to –0.9)	–0.8 (–1.3 to –0.4)	<.001*^b^*
26 wk	42/47	7.5 (2.0)	9.0 (1.7)	–1.5 (–2.3 to –0.7)	–0.8 (–1.2 to –0.4)	<.001*^b^*
52 wk	41/46	6.7 (2.8)	8.7 (1.8)	–1.9 (–2.9 to –0.9)	–0.8 (–1.2 to –0.4)	<.001*^b^*
General health (from EuroQol thermometer, 0-100 scale): higher scores indicate better general health						
Baseline	50/50	79.2 (11.5)	76.7 (13.3)	—	—	—
4 wk	50/50	80.3 (12.0)	80.6 (14.4)	–2.7 (–7.2 to 1.8)	0.0 (–0.4 to 0.4)	.238
12 wk	47/50	83.6 (12.1)	84.2 (9.5)	–3.6 (–8.9 to 1.6)	–0.3 (–0.7 to 0.1)	.174
26 wk	42/47	81.1 (14.1)	83.5 (11.0)	–4.7 (–10.3 to 1.0)	–0.4 (–0.8 to 0.1)	.103
52 wk	41/46	81.0 (15.5)	85.9 (7.9)	–8.1 (–13.9 to –2.4)	–0.7 (–1.1 to –0.2)	.006*^b^*
EQ-5D-5L (5-item questionnaire used to derive utility score between –0.280 and 1.0), with higher scores indicating greater quality of life						
Baseline	50/50	0.77 (0.08)	0.75 (0.10)	—	—	
4 wk	50/50	0.80 (0.09)	0.77 (0.08)	0.00 (–0.05 to 0.04)	–0.05 (–0.44 to 0.35)	.852
12 wk	47/50	0.84 (0.13)	0.84 (0.12)	–0.01 (–0.06 to 0.04)	–0.06 (–0.46 to 0.33)	.762
26 wk	42/47	0.84 (0.12)	0.84 (0.11)	–0.02 (–0.07 to 0.04)	–0.15 (–0.56 to 0.27)	.526
52 wk	41/46	0.84 (0.13)	0.84 (0.11)	–0.03 (–0.09 to 0.02)	–0.27 (–0.69 to 0.15)	.230

a SMD, standardized mean difference; Victorian Institute of Sport Assessment Scale for Proximal Hamstring Tendinopathy. Dashes indicate not applicable.

b Significant between-group difference.

**Table 4:** Effects of Physiotherapy Versus Shockwave on Ordinal Primary and Secondary Outcomes

		Median (25th-75th percentile)	
Outcome	No. Included, PHYSIOTHERAPY/SHOCKWAVE	PHYSIOTHERAPY	SHOCKWAVE	*P* Value
Global rating of change (1-7 scale, with lower scores indicating greater improvement)				
4 wk	50/50	3.0 (2.0-4.0)	3.0 (2.0-3.0)	.437
12 wk	47/50	2.0 (2.0-3.0)	2.0 (2.0-3.0)	.832
26 wk	44/47	2.0 (2.0-3.0)	2.0 (2.0-3.0)	.456
52 wk	42/46	2.0 (2.0-3.0)	2.0 (1.0-3.0)	.797
Satisfaction with treatment (1-5 scale, with lower scores indicating greater satisfaction)				
4 wk	50/50	2.0 (1.0-2.0)	1.5 (1.0-2.0)	.674
12 wk	48/49	1.0 (1.0-2.0)	1.0 (1.0-2.0)	.962
26 wk	42/47	2.0 (1.0-2.0)	1.0 (1.0-2.0)	.028*^a^*
52 wk	41/46	2.0 (1.0-2.0)	1.0 (1.0-2.0)	.238
Satisfaction with results of treatment (1-5 scale, with lower scores indicating greater satisfaction)				
4 wk	50/50	2.0 (2.0-3.0)	2.0 (1.0-2.0)	.014*^a^*
12 wk	47/50	2.0 (1.0-3.0)	2.0 (1.0-3.0)	.617
26 wk	42/47	2.0 (2.0-3.0)	2.0 (1.0-3.0)	.046*^a^*
52 wk	41/46	2.0 (1.0-3.0)	2.0 (1.0-3.0)	.521
Physical Activity Level Scale (1-6 scale, with higher scores indicating higher levels of physical activity)				
Baseline	50/50	5.5 (5.0-6.0)	5.0 (4.0-6.0)	
4 wk	50/50	5.0 (4.0-6.0)	5.0 (5.0-6.0)	.919
12 wk	47/50	6.0 (5.0-6.0)	5.0 (4.0-6.0)	.331
26 wk	43/47	6.0 (5.0-6.0)	6.0 (5.0-6.0)	.799
52 wk	41/46	6.0 (5.0-6.0)	5.0 (5.0-6.0)	.933
Sitting symptoms (1-5 scale, with lower scores indicating higher sitting tolerance)				
Baseline	50/50	2.0 (1.0-3.0)	2.0 (1.0-3.0)	
4 wk	50/50	1.0 (1.0-2.0)	1.0 (1.0-2.0)	.772
12 wk	47/50	1.0 (1.0-1.0)	1.0 (1.0-1.0)	.812
26 wk	43/47	1.0 (1.0-2.0)	1.0 (1.0-2.0)	.808
52 wk	41/46	1.0 (1.0-1.0)	1.0 (1.0-2.0)	.276

a Significant between-group difference.

For continuous secondary outcome data (Table 3), the SHOCKWAVE intervention demonstrated a significantly lower Örebro Musculoskeletal Pain Screening Questionnaire–Short Form (ÖMPSQ-SF) score at 26 weeks (mean difference, 4.7 [95% CI, 0.2 to 9.1]; *P* = .039) and better general health (measured by the EQ-5D-5L thermometer) at 52 weeks (mean difference, 8.1 [95% CI, –13.9 to –2.4]; *P* = .006). Participant self-rated adherence was significantly higher for the SHOCKWAVE intervention at all time points (12-week mean difference, 1.6 [95% CI, –2.4 to –0.9]; *P* < .001).

For ordinal secondary outcome data (Table 4), the SHOCKWAVE intervention demonstrated a significantly higher satisfaction with treatment at 26 weeks, and a significantly higher satisfaction with the results of treatment at 4 and 26 weeks.

A responder analysis showed no significant between-group differences in the proportion of participants who achieved clinically important changes on the VISA-H or GROC (Table 5). Within-group analyses of the VISA-H showed statistically (at all time points) and clinically (at 12, 26, and 52 weeks) significant improvement from baseline for both interventions.

**Table 5:** Proportion of Participants in Each Treatment Group With Clinically Important Changes in Outcomes*^a^*

	Proportion of Participants Achieving Clinically Important Change					
Outcome	PHYSIOTHERAPY	SHOCKWAVE	Risk Difference (95% CI), %	Relative Risk (95% CI)	*P* Value	NNT (95% CI)
Global rating of change “much improved” or “completely recovered”						
4 wk	16/50	17/50	2 (–16 to 20)	1.1 (0.6 to 1.9)	.832	–50 (–5 to 6)
12 wk	27/47	28/50	–1 (–20 to 18)	1.0 (0.7 to 1.4)	.886	69 (–6 to 5)
26 wk	27/44	29/47	00 (19 to 20)	1.0 (0.7 to 1.4)	.974	–295 (–5 to 5)
52 wk	26/42	27/46	–3 (–23 to 17)	0.9 (0.7 to 1.3)	.759	31 (–6 to 4)
Reduced VISA-H score of at least 12 points from baseline						
4 wk	19/50	19/50	00 (–18 to 18)	1.0 (0.6 to 1.6)	1	NA (–5 to 5)
12 wk	26/49	31/50	9 (–10 to 27)	1.2 (0.8 to 1.6)	.368	–11 (–4 to 10)
26 wk	29/43	37/47	11 (–7 to 29)	1.2 (0.9 to 1.5)	.227	–9 (–3 to 14)
52 wk	32/42	37/46	4 (–13 to 21)	1.1 (0.8 to 1.3)	.629	–24 (–5 to 8)

a NA, not applicable; NNT, number needed to treat; VISA-H, Victorian Institute of Sport Assessment Scale for Proximal Hamstring Tendinopathy.

Given the differences in symptom duration at baseline between the 2 intervention groups, an additional analysis of the VISA-H was undertaken controlling for duration of symptoms (Appendix Table A2, available online); however, this did not show any meaningful differences compared with the initial analysis.

## Discussion

There was no difference in effectiveness between a 6-session SHOCKWAVE intervention and a 6-session PHYSIOTHERAPY intervention for participants with PHT in primary outcome measures of VISA-H and GROC. Some secondary outcomes favored SHOCKWAVE over PHYSIOTHERAPY: ÖMPSQ-SF (26 weeks), general health (52 weeks), satisfaction with treatment (26 weeks), and satisfaction with results of treatment (4 and 26 weeks), but the size of the effects may not be clinically important. Additionally, participant self-rated adherence was higher in the SHOCKWAVE group at all time points. Trial feasibility measures were met for participant retention and completion (>85%), but not for recruitment rate (6 per month).

The use of 2 active intervention groups may partly explain the lack of detected differences in outcomes. SHOCKWAVE and PHYSIOTHERAPY are both plausible interventions that could both produce a therapeutic effect, albeit via different mechanisms. In addition, both groups received the same education about managing PHT, which could potentially be effective on its own. Working alliance between the participants and the treating physiotherapists may have also contributed to the improvement in both groups.^60,61^ With this trial design, we were unable to blind patients and therefore unable to determine whether therapeutic treatment effects were greater than nonspecific effects, such as placebo or natural history, although with a mean condition duration of 110 weeks, a strong natural recovery rate would be unexpected. This could be a direction of future research. However, recent trials comparing ESWT to sham^4,63,90,92^ and exercise to sham^32^ in other tendinopathies have failed to demonstrate superiority of these interventions over nonspecific effects. There is clearly a need for more research to determine whether any treatments are more effective than placebo and other interventions for PHT and other tendinopathies.

The differences in the participant self-rating of adherence between the 2 interventions are likely due to the higher demands in the PHYSIOTHERAPY intervention, with participants generally expected to undertake approximately 2 hours of rehabilitation exercise per week during and after the intervention. This may be an important finding in favor of the feasibility of SHOCKWAVE over PHYSIOTHERAPY and could form part of treatment selection considerations by therapists and patients.

The differences seen in satisfaction with treatment (26 weeks only) and satisfaction with results of treatment (4 and 26 weeks only) may have been incidental findings given that they were only observed at 1 to 2 individual time points. The use of nonparametric testing for analyzing satisfaction data also provided less precision and power than in the analysis of continuous outcomes.

There are different approaches to rehabilitation-based interventions for managing tendinopathy. No research has demonstrated which rehabilitation approach is most effective for PHT, although clinical and research tendinopathy experts recommend similar programs to the one applied in this trial,^34,59,69^ and similar approaches have been successful in other tendinopathy trials.^#^ An advantage of the rehabilitation program used in this trial was that it could be undertaken at a location convenient to the participant, with minimal equipment. More than 6 sessions of rehabilitation may have potentially yielded more effective results, and further research is needed to confirm optimal treatment parameters for PHT rehabilitation.

Imaging studies were not used to assist with forming a diagnosis for participants in this trial. There are no gold-standard diagnostic tests for PHT, and pathological changes in asymptomatic people are known to be common^18^; however, exclusion of potential participants with no changes on MRI may have increased confidence of PHT being the cause of symptoms. Imaging for potential participants was a cost beyond the scope of this trial, and the thorough telephone screening and clinical examination used should have reduced inclusion of inappropriate participants in this trial. It is, however, acknowledged that some uncommon neural causes of lower buttock pain (eg, local sciatic nerve or posterior femoral cutaneous nerve neuropathies) may have benefitted from the hypothesized neuropraxic and analgesic effects of shockwave treatment (and would be unlikely to benefit from a rehabilitation program), although these conditions were screened for during eligibility assessment. While other psychological variables were measured in this trial in line with core domains for tendinopathy studies,^94^ self-efficacy was not measured. Rehabilitation-based tendinopathy interventions are hypothesized to increase self-efficacy,^60^ and increased self-efficacy was reported by participants in the PHYSIOTHERAPY intervention during exit interviews (manuscript in preparation). Measurement of this variable could have provided further insights into the benefits of advice and rehabilitation.

The involvement of 26 physiotherapists across 10 clinics in this trial increases generalizability of results but could introduce inconsistencies between clinicians with adherence to the intervention protocols. The participants in this trial were largely recreational distance runners, mostly female (74%), with a mean age of 45 years. While they were generally still highly active, this cohort does not have high-energy storage and release demands for their hamstring tendon complex, such as in sports involving sprinting or picking up a ball off the ground while running. This is in contrast to the RCT of Cacchio et al,^10^ which investigated mostly male professional athletes (68%) with a mean age of 24 years. There may be different responses to a rehabilitation-based intervention such as the one in this trial in different cohorts, and this could be the target of future trials.

Based on the results of this RCT, which met most feasibility aims but demonstrated no statistically or clinically significant between-group differences in primary outcome measures at any time point, there is no justification for proceeding with a fully powered RCT at this stage. While future trials on PHT appear feasible, focusing on different intervention and/or comparison groups, or a different sample population, may be more fruitful to determine the most effective treatments for this condition.

## Conclusion

This is the first trial to investigate an individualized physiotherapy program for PHT using principles successful with other tendinopathies. While the trial met most feasibility thresholds, there were no differences between physiotherapy and shockwave for the primary outcomes of global effect or function.

## Supplemental Material

sj-docx-1-ajs-10.1177_03635465251391134 – Supplemental material for Physiotherapy Compared With Shockwave Therapy for the Treatment of Proximal Hamstring Tendinopathy: A Randomized Controlled Trial Supplemental material, sj-docx-1-ajs-10.1177_03635465251391134 for Physiotherapy Compared With Shockwave Therapy for the Treatment of Proximal Hamstring Tendinopathy: A Randomized Controlled Trial by Aidan Rich, Jon Ford, Jilliane Cook and Andrew Hahne in The American Journal of Sports Medicine

## Footnotes

∥ References 1, 2, 17, 19, 23, 33, 35, 45, 56, 71, 82, 85, 89, 99.

¶ References 3, 12, 24, 28, 30, 40, 44, 62, 73, 74, 96,

# References 1, 2, 17, 19, 23, 33, 35, 45, 56, 71, 82, 85, 89, 99.
