# Supplementary material for: Physiotherapy Compared With Shockwave Therapy for the Treatment of Proximal Hamstring Tendinopathy: A Randomized Controlled Trial
Source: Am J Sports Med. 2025 Nov 16;53(14):3396–407. doi: 10.1177/03635465251391134 (PMC12657663; doi:10.1177/03635465251391134)
Supplement: sj-docx-1-ajs-10.1177_03635465251391134 – Supplemental material for Physiotherapy Compared With Shockwave Therapy for the Treatment of Proximal Hamstring Tendinopathy: A Randomized Controlled Trial [file sj-docx-1-ajs-10.1177_03635465251391134.docx]

APPENDIX TABLE A1

Eligibility Criteria*^a^*

| Inclusion Criteria |
| --- |
| Initial phone screening |
| 1. Reports of relatively localized (defined as an area smaller than a tennis ball) ischial tuberosity region pain^34^ of gradual onset and at least 3 mo in duration |
| 1. Willingness to participate in 6 sessions of intervention over a 12-wk period |
| 1. Age between 18 and 65 y, inclusive |
| 1. Fluency in English sufficient to complete questionnaires and to enable understanding of the intervention 2. Agreeing to refrain from other interventions for the treatment period of the trial, aside from consultation with medical practitioners and medication |
| Clinical examination screening |
| 1. A history of increased tendon load precipitating the onset of symptoms determined based on clinical interview |
| 1. Reproduction of ischial tuberosity region pain with ≥3 of the following loading/compressive tests:  - Single-leg arabesque - Supine single-leg bridge with heel on standardized height platform (bent knee) - Self-reported PHT symptoms with prolonged sitting for <30 min - Modified bent-knee hamstring stretch test^8^ |
| Exclusion Criteria |
| Initial phone screening |
| 1. Previous surgery to the hamstring complex, as we wish to study treatment effects independent of the effects of surgical procedures |
| 1. Previous injection to the hamstring tendon within the previous 6 wk, as we wish to study treatment effects independent of the effects of injections |
| 1. Treatment with ESWT for PHT in the last 3 mo,^88^ as this may influence progress during the trial |
| 1. Contraindications to receiving ESWT^21^ |
| 1. Current pregnancy or recent childbirth (within 6 mo), as this could impair ability to undertake testing and intervention |
| 1. Diagnosis with autoimmune disease, as these conditions may have a negative impact on tendon |
| 1. Already received >2 sessions of physiotherapy with any of the trial physiotherapists before enrollment, as these therapists are likely to use components of the trial treatment protocol on their clinical caseload |
| 1. An active compensation claim for the injury, as this may influence the response to treatment^67^ |
| 1. Planned absence for a period of >2 wk during the treatment period (such as extended holiday) |
| Clinical examination screening |
| 1. Pain that is predominantly due to lumbar dysfunction, including lumbar spine radiculopathy,^95^ or lumbar spine somatic referral |
| 1. Pain that is reasoned from clinical examination to be predominantly due to other structures or conditions, including sciatic nerve entrapment, ischiofemoral impingement, hip joint, local sciatic nerve irritation, and adductor magnus tendinopathy |

*^a^*ESWT, extracorporeal shockwave therapy; PHT, proximal hamstring tendinopathy.

APPENDIX TABLE A2

Effects of Physiotherapy Versus Shockwave on VISA-H (adjusted for the covariate of symptom duration)*^a^*

| Outcome | No. Included,  PHYSIOTHERAPY/SHOCKWAVE | Unadjusted Mean Score (SD) | | Adjusted Between-Group Difference (95% CI) | Adjusted SMD (95% CI) | *P* Value |
| --- | --- | --- | --- | --- | --- | --- |
|  |  | PHYSIOTHERAPY | SHOCKWAVE |  |  |  |
| Pain, function, and sporting activity (VISA-H: 8-item questionnaire scored out of 100): higher scores indicate lower pain, higher function, and sporting activity | | | | | | |
| Baseline | 50/50 | 49.8 (15.4) | 50.7 (11.8) |  |  |  |
| 4 wk | 50/50 | 57.6 (16.1) | 56.5 (15.5) | 2.0 (–4.3 to 8.3) | 0.1 (–0.3 to 0.5) | .529 |
| 12 wk | 47/50 | 65.2 (16.9) | 65.5 (16.4) | 0.8 (–5.5 to 7.2) | 0.1 (–0.3 to 0.5) | .795 |
| 26 wk | 43/47 | 68.3 (17.9) | 72.8 (15.1) | –3.7 (–10.3 to 2.7) | –0.2 (–0.6 to 0.2) | .259 |
| 52 wk | 41/46 | 73.9 (18.9) | 74.7 (17.9) | –0.4 (–7.0 to 6.2) | 0.0 (–0.4 to 0.4) | .912 |

*^a^*SMD, standardized mean difference; VISA-H, Victorian Institute of Sport Assessment Scale for Proximal Hamstring Tendinopathy.
